# Incretin-Based Therapy for Prevention of Diabetic Vascular Complications

**DOI:** 10.1155/2016/1379274

**Published:** 2015-12-31

**Authors:** Akira Mima

**Affiliations:** Department of Nephrology, Nara Hospital, Kindai University Faculty of Medicine, Nara 630-0293, Japan

## Abstract

Diabetic vascular complications are the most common cause of mortality and morbidity worldwide, with numbers of affected individuals steadily increasing. Diabetic vascular complications can be divided into two categories: macrovascular andmicrovascular complications. Macrovascular complications include coronary artery diseaseand cerebrovascular disease, while microvascular complications include retinopathy and chronic kidney disease. These complications result from metabolic abnormalities, including hyperglycemia, elevated levels of free fatty acids, and insulin resistance. Multiple mechanisms have been proposed to mediate the adverse effects of these metabolic disorders on vascular tissues, including stimulation of protein kinase C signaling and activation of the polyol pathway by oxidative stress and inflammation. Additionally, the loss of tissue-specific insulin signaling induced by hyperglycemia and toxic metabolites can induce cellular dysfunction and both macro- and microvascular complications characteristic of diabetes. Despite these insights, few therapeutic methods are available for the management of diabetic complications. Recently, incretin-based therapeutic agents, such as glucagon-like peptide-1 and dipeptidyl peptidase-4 inhibitors, have been reported to elicit vasotropic actions, suggesting a potential for effecting an actual reduction in diabetic vascular complications. The present review will summarize the relationship between multiple adverse biological mechanisms in diabetes and putative incretin-based therapeutic interventions intended to prevent diabetic vascular complications.

## 1. Introduction

The number of patients suffering from diabetes worldwide is rapidly increasing. A recent report prepared by the international diabetes foundation (IDF) estimates the global number of patients with diabetes to have risen to 380 million, with the total number of patients predicted to reach 590 million by 2035. Furthermore, the majority of new diabetic patients come from Southeast Asia and west Pacific regions (http://www.idf.org/). Diabetes-induced macro- and microvascular complications and their pathologies are the major contributors to morbidity and mortality. Macrovascular complications of diabetes involve large vessel obstructions, including peripheral artery disease, coronary artery disease, atherosclerosis, and cerebrovascular disease, while microvascular pathologies include retinopathy, neuropathy, and nephropathy. Abnormal metabolites formed in hyperglycemic state are major systemic risk factors for these diabetic complications. The Diabetes Control and Complications Trial (DCCT) performed in type 1 diabetic patients and United Kingdom Prospective Diabetes Study (UKPDS) in type 2 diabetic patients clearly showed that intensive glycemic control could delay the onset of diabetes and retard the occurrence of diabetic complications [1, 2]. Furthermore, the follow-up of both DCCT and UKPDS trials showed that such intensive glycemic control could decrease diabetic macrovascular complications [3, 4]. Hyperglycemia could therefore be a major factor for initiation and progression of diabetic complications. However, hyperglycemia alone is not enough to induce such vascular complications; multiple potential biochemical pathways have been proposed to underlie the adverse effects of diabetes-induced vascular complications. Activation of diacylglycerol- (DAG-) protein kinase C (PKC) signaling, increased oxidative stress and inflammation, enhanced polyol pathway, activation of the hexosamine pathway, and overproduction of advanced glycation end products (AGEs) have all been proposed as potential intra- and extracellular changes which lead to alterations in the signaling pathways associated with vascular complications in diabetes [5–7]. While a study by Coca et al. has demonstrated that intensive glycemic control reduces albuminuria, there is currently no evidence that would suggest that intensive glycemic control reduces the risk of renal outcomes [8]. Additionally, findings of other groups suggest that hypoglycemia exacerbates both macrovascular and microvascular complications of diabetes and increases the risk of morbidity and mortality [9]. On the basis of these published findings, intensive lowering of blood glucose may be less beneficial for diabetes-induced vascular complications.

Incretins are a family of gut hormones which includes glucose-dependent insulinotropic polypeptide (GIP) and glucagon-like peptide-1 (GLP-1) [10]. Furthermore, a number of recent studies indicate that GLP-1 and dipeptidyl peptidase-4 (DPP-4) inhibitors exhibit potent pleiotropic protective effects on diabetic vascular complications, beyond their effects on glycemic control [11, 12]. One specific feature of incretin hormone-related drugs is a reduced danger of hypoglycemia [13]. Therefore, incretin-based therapeutic agents could prevent and slow the progression of diabetic vascular complications.

To elucidate these concepts, we will briefly review the multiple biochemical pathways and novel knowledge gathered about incretins, selecting the literature describing incretin-related pathways as therapeutic targets for the management of diabetic vascular complications.

## 2. Inflammation, Oxidative Stress, and PKC Activation in Diabetic Vascular Complications

Increasing the formation of aggressive T cells and altering the Th1/Th2 cell ratio towards the proinflammatory status have been reported to lead to the initiation and progression to overt diabetes [14]. In spite of C-reactive protein (CRP) levels in patients with recent-onset type 1 diabetes being the same as those measured in the control group, elevated CRP levels have been observed in long-term type 1 diabetes patients [15]. High glucose levels increase interleukin- (IL-) 12 production in macrophages and interferon-*γ* production in CD4^+^ T cells [16]. Additionally, hyperglycemia-induced activation of nuclear factor-*κ* (NF-*κ*) B increases the levels of endothelin-1 (ET-1), vascular cell adhesion molecule- (VCAM-) 1, intercellular adhesion molecule- (ICAM-) 1, IL-6, and tumor necrosis factor- (TNF-) *α* [11, 17]. Furthermore, a positive relationship has been demonstrated between plasma interferon- (IFN-) *γ*, estimated glomerular filtration rate (eGFR), and proteinuria [18]. Therefore, anti-inflammatory drugs could provide useful new approaches for the management of diabetic vascular complications. Despite favorable results reported in rodent models, it is still unclear whether anti-inflammatory drugs, including adiponectin, NF-*κ*B inhibitors, COX2 inhibitors, and inhibitors of chemokine C-X-C motif ligand 2 (CXCL2), can elicit significant effects against diabetic vascular complications in humans.

Activation of DAG-PKC signal transduction pathway was shown to be related to diabetic microvascular diseases, with increases in PKC activity known to induce extracellular matrix (ECM) accumulation, epithelial cell apoptosis, monocyte adhesion, and cytokine activation [19]. PKC induces oxidative stress by activating the mitochondrial NADPH oxidase [20, 21]. Additionally, there is evidence that oxidants and AGEs can increase DAG levels and activate PKC [5]. Altered levels of reactive oxygen species (ROS) have been reported in the kidneys and retina of both animal models of diabetes and in patients [17, 22–25]. Also, increased plasma levels of 8-hydroxydeoxyguanosine (8-OHdG) and lipid peroxides have been reported to result from abnormal metabolism of glucose and free fatty acids [26–28].

In a phase II clinical trial in US, ruboxistaurin (RBX), a PKC*β* isoform selective inhibitor, decreased albuminuria significantly and did not show increases of urinary TGF-*β*. Furthermore, RBX-treated patients maintained a stable eGFR over 1 year [29]. Administration of antioxidant agents, including vitamins C and E, has been evaluated in murine models of diabetes. In most studies, administration of these drugs was shown to effectively ameliorate the pathological changes in murine models of diabetic nephropathy [30–32]. Additionally, other studies have demonstrated the effectiveness of vitamin E administration in normalizing oxidative stress markers and decreasing PKC-induced diabetic vascular complications. Despite the favorable results observed in animal studies, in the Heart Outcomes Prevention Evaluation (HOPE) study evaluating a large cohort of patients with diabetes, administration of vitamin E did not reduce the risk of cardiovascular complications [30]. It is possible that the plasma levels of these vitamins might not reflect those at the tissue levels. Lastly, bardoxolone methyl, a new antioxidant drug, interacts with the cysteine residues on Keap1 to cause the translocation of nuclear factor erythroid 2-related factor 2 (Nrf2) to the nucleus, increasing its anti-inflammatory effects [33–35]. This drug was expected to ameliorate diabetic nephropathy on the basis of beneficial effects observed in recent placebo-controlled clinical trials [36, 37]. However, due to high mortality observed in the treated group, this phase 3 trial was suspended. The effectiveness of antioxidant therapies in management of diabetes therefore remains unknown.

## 3. Incretin: A New Class of Antidiabetic Drugs

Incretins are a family of gut hormones and released from the gut in response to ingestions of various nutrients [38]. GIP and GLP-1 could induce biological effects through the GIP receptors (GIPR) and GLP-1 receptors (GLP-1R). GLP-1 binds to GLP-1R in the vascular endothelial cells [39–41]. GLP-1 also binds to GLP-1R in intestinal mucosa and the portal vein to induce insulin secretion using the nervous system [42]. GLP-1 suppresses inflammatory markers, such as CD68, CXCL2, and plasminogen activator inhibitor- (PAI-) 1, and this effect could be involved in the cAMP/protein kinase A (PKA) pathway. Like GLP-1, GIP could inhibit production of reactive oxygen species (ROS) as well as PAI-1 via the cAMP pathway [43]. In addition to its effect on insulin and glucagon, GLP-1 exhibits a number of vasotropic actions beyond glycemic control; it increases insulin sensitivity in peripheral tissues, improves endothelial function, and decreases inflammation in some organs [41, 44]. Both GIP and GLP-1 are easily degraded by DPP-4, terminating biological effects. Inhibition of DPP-4 increases circulating GLP-1 levels and was shown to be a useful intervention in type 2 diabetic patients [13, 45, 46]. DPP-4 inhibitors could also reduce inflammation, with decreased levels of MCP-1 reported following administration of DPP-4 inhibitors [47]. Like GLP-1, DPP-4 inhibitors also elicit vasotropic effects and can potentially be used for the amelioration of diabetic nephropathy [48, 49].

## 4. The Effects of Incretin on Inflammation and Oxidative Stress

Anti-inflammatory and antioxidative stress effects of incretin have been described previously. In endothelial cells, GLP-1 reduces TNF-*α* expression and ROS production, inhibiting the adhesion and activation of macrophages [50]. Exenatide, a GLP-1R agonist, has been reported to significantly attenuate mRNA levels of MCP-1 and TNF-*α*, decreasing atherosclerosis [51]. Additionally, we have reported that administration of GLP-1 decreases diabetes-induced inflammation and oxidative stress in the glomerulus [11].

## 5. Renal Effects of Incretin

Diabetic nephropathy (DN) is one of the major diabetic microvascular complications, leading to chronic kidney disease (CKD) [52]. According to United States Renal Data System (USRDS), up to 44% of patients with type 2 diabetes in the United States develop overt DN (USRDS 2007 Annual Data Report. Bethesda, MD: National Institute of Diabetes and Digestive and Kidney Diseases, National Institutes of Health, U.S. Department of Health and Human Services; 2007).

GLP-1 has been reported to elicit renal protective effects against DN [11, 54]. Specifically, GLP-1 decreases inflammatory and oxidative stress markers in glomerular endothelial cells [11, 55].

**Figure 1:**
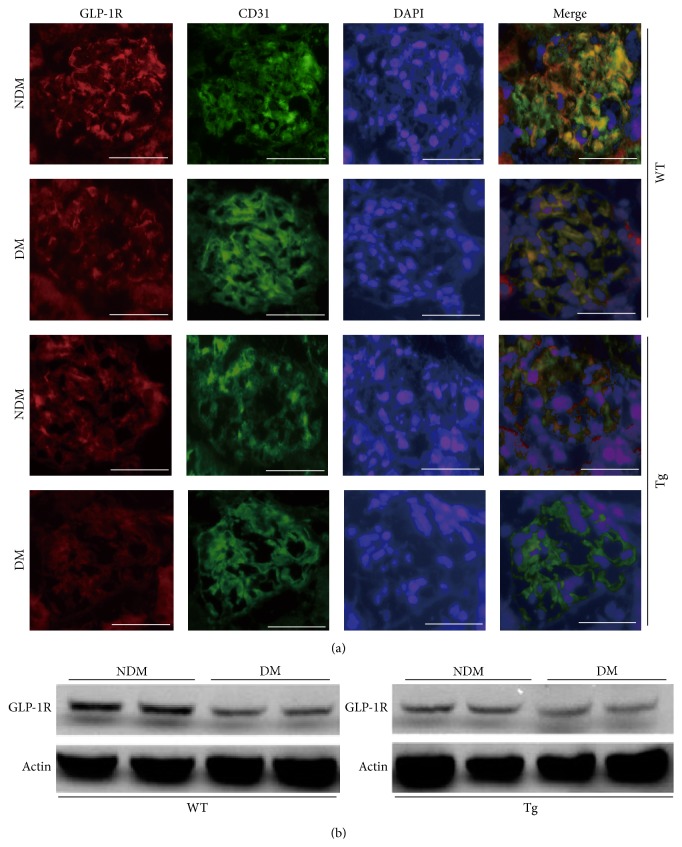
Overexpression of PKC*β*2 in mouse glomerular endothelial cells (EC-PKC*β*2Tg) and decreased glucagon-like peptide-1 receptors in diabetes. (a) Immunostaining of GLP-1R and CD31, showing merged images of the glomeruli. (b) Immunoblots of GLP-1R from renal cortex of mice. Images are reproduced from Mima et al. [11], with permission from Diabetes ©2012.

**Figure 2:**
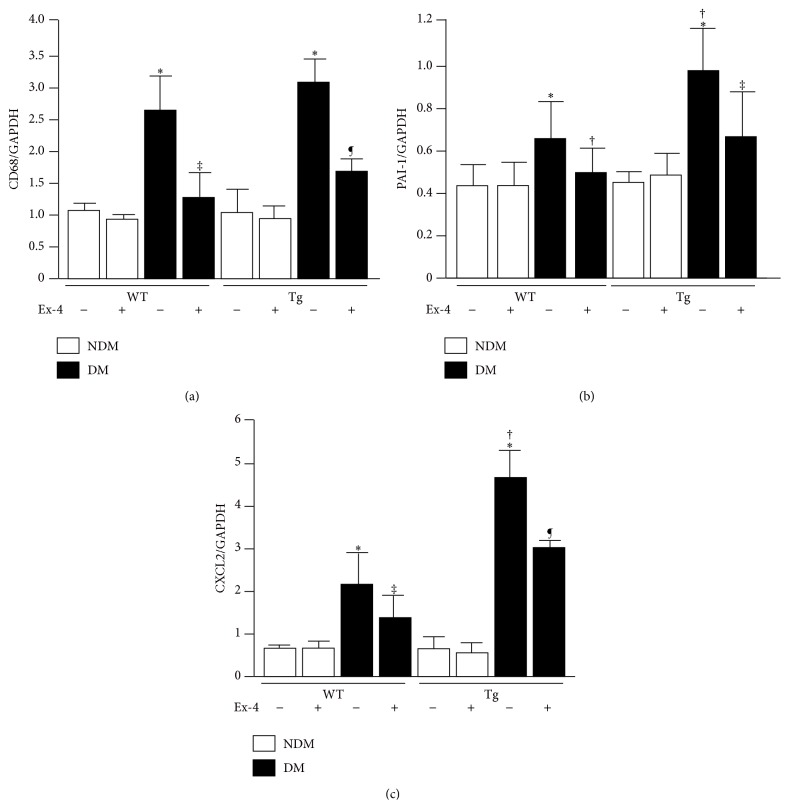
Effect of exendin-4 treatment on inflammatory markers in EC-PKC*β*2Tg mice. (a) CD68 mRNA expression in the renal cortex of each group. ^*∗*^ *P* < 0.05 versus WT/NDM/exendin-4(−), ^†^ *P* < 0.05 versus WT/DM/exendin-4(−), and ^‡^ *P* < 0.05 versus EC-PKC*β*2Tg/DM/exendin-4(−). *N* = 6 in nondiabetic WT + vehicle, nondiabetic WT + Ex-4, diabetic WT + vehicle, diabetic WT + Ex-4, nondiabetic EC-PKC*β*2Tg + Ex-4, and diabetic EC-PKC*β*2Tg + Ex-4 groups; *n* = 7 in nondiabetic EC-PKC*β*2Tg + vehicle and diabetic EC-PKC*β*2Tg + vehicle groups. (b) PAI-1 mRNA expression in the renal cortex of each group. ^*∗*^ *P* < 0.05 versus WT/NDM/exendin-4(−), ^†^ *P* < 0.05 versus WT/DM/exendin-4(−), and ^‡^ *P* < 0.05 versus EC-PKC*β*2Tg/DM/exendin-4(−). *N* = 6 in nondiabetic WT + vehicle, nondiabetic WT + Ex-4, diabetic WT + vehicle, diabetic WT + Ex-4, nondiabetic EC-PKC*β*2Tg + Ex-4, and diabetic EC-PKC*β*2Tg + Ex-4 groups; *n* = 7 in nondiabetic EC-PKC*β*2Tg + vehicle and diabetic EC-PKC*β*2Tg + vehicle groups. (c) CXCL2 mRNA expression in the renal cortex of each group. ^*∗*^ *P* < 0.05 versus WT/NDM/exendin-4(−), ^†^ *P* < 0.05 versus WT/DM/exendin-4(−), and ^‡^ *P* < 0.05 versus EC-PKC*β*2Tg/DM/exendin-4(−). *n* = 6 in nondiabetic WT + vehicle, nondiabetic WT + Ex-4, diabetic WT + vehicle, diabetic WT + Ex-4, nondiabetic EC-PKC*β*2Tg + Ex-4, and diabetic EC-PKC*β*2Tg + Ex-4 groups; *n* = 7 in nondiabetic EC-PKC*β*2Tg + vehicle and diabetic EC-PKC*β*2Tg + vehicle groups. Reproduction from Mima et al. [11] with permission from Diabetes ©2012.

In kidney, GLP-1R were mainly detected in renal glomeruli but not in tubules. Recent studies highlighted the lack of specificity of multiple anti-GLP-1R antibody [51, 63]. However, Fujita et al. clearly showed the presence of GLP-1R in renal glomeruli using* in situ *hybridization, supporting previous reports [64]. We have shown that exendin-4, a GLP-1R agonist, activates the cAMP/PKA signaling pathway, resulting in increased phospho-c-Raf (Ser259) levels which could inhibit phospho-c-Raf (Ser338)/phospho-Erk1/2/PAI-1 signaling activated by angiotensin II or PKC*β* [11]. Interestingly, PKC*β* increases the levels of ubiquitinated GLP-1R, decreasing GLP-1R protein levels in the glomeruli (Figure 1). In contrast, PKC*α*, another PKC isoform, decreases RNA levels of GLP-1R in the pancreas [65]. Additionally, we showed that exendin-4 decreased the mRNA levels of inflammatory markers CD68, PAI-1, and CXCL2 in the renal cortex of diabetic rodents [11] (in diabetic WT mice, decreased by 52 ± 7, 24 ± 9, and 36 ± 11%, resp.; Figure 2). Other researchers have demonstrated the presence of the same mechanism in mesangial cells, showing GLP-1-induced increases in cAMP to suppress the inflammatory response against AGEs by decreasing the expression of receptor for AGEs (RAGE) [66]. GLP-1 decreases albuminuria and ameliorates mesangial expansion, which is a typical pathological feature of DN [11, 54]. 8-OHdG and malondialdehyde (MDA), markers of oxidative stress, are significantly increased in diabetic and insulin-resistant conditions [55]. Administration of GLP-1R agonist liraglutide was shown to decrease the levels of these oxidative stress markers and ameliorate renal function in diabetic rats [56]. GLP-1 directly suppresses transforming growth factor- (TGF-) *β* signal, which is related to glomerular injury, mesangial matrix expansion, and increasing extracellular matrix in DN [55]. Furthermore, the results from the Akita* Glp1r* ^−/−^ mice showed severe mesangial expansion and increases in glomerular ROS, upregulated renal NADPH oxidase, and decreased renal cAMP/PKA activity [64]. Taking these reports together, GLP-1 may have renoprotective effects at least in rodent DN models.

While a relatively small number of studies have demonstrated the effectiveness of GLP-1 therapy on human DN, intravenous infusion of GLP-1 in insulin-resistant states was shown to increase sodium excretion, reduce H^+^ secretion, and reduce glomerular hyperfiltration, leading to renoprotective effects [67]. Interestingly, our recent findings show that GLP-1R protein levels were decreased in the renal cortex of patients with type 1 diabetes of extreme duration (Mima A, King GL, unpublished observation).

## 6. Renal Effects of DPP-4 Inhibitors

Several DPP-4 inhibitors were reported to ameliorate renal function and pathology in DN. Linagliptin has been reported to decrease renal fibrosis following diabetes-induced endothelial-to-mesenchymal transition through an effect mediated by microRNA 29s [57]. Vildagliptin was reported to elicit renoprotective effects, such as a reduction in albuminuria [68] and decreased ECM deposition in the diabetic glomeruli by reducing the levels of DPP-4 and increasing levels of GLP-1 [69]. Furthermore, as we have previously shown using GLP-1R agonist exendin-4 [11], vildagliptin decreased the oxidative stress in the kidney of rat model of type 2 diabetes [70]. Additionally, sitagliptin ameliorated diabetes-induced renal pathological changes accompanied by decreased lipid peroxidation [71]. In patients with type 2 diabetes, sitagliptin and alogliptin reduced albuminuria and 8-OHdG, markers of oxidative stress, and increased urinary cAMP levels, leading to renoprotective effects [49]. Recent subanalysis study of SAVOR-TIMI 53 shows that saxagliptin significantly decreases albuminuria in both microalbuminuria and overt proteinuria state of type 2 DN patients (Frederich B et al. presentation abstract; American Diabetes Association 74th Scientific Sessions, 2014). Furthermore, the MARLINA-T2D trial will examine the effect of linagliptin on albuminuria in people with type 2 diabetes [53] (Table 1). However, it seems that the renoprotective effects of DPP-4 inhibitors found in most studies depend on the amelioration of glycemic control or increased GLP-1 levels. Therefore, further studies are needed to clarify the effects of DPP-4 inhibitors on DN.

## 7. Cardiovascular Effects of Incretin-Based Therapeutic Agents

A number of studies have demonstrated the effects of incretins on macrovascular complications, including coronary artery disease (CAD), atherosclerosis, and cerebrovascular disease [72]. Intravenous administration of GLP-1 reduced infarct size after the occlusion of left anterior descending coronary artery in rats [73]. Administration of liraglutide could increase the expression of cardioprotective genes, leading to beneficial effects on cardiac tissues [12]. Additionally, GLP-1 treatment has been reported to significantly reduce infarct size and improve cardiac function in pigs [74]. We have shown that GLP-1 incubation increases cAMP and PKA levels in glomerular endothelial cells [11]. Similarly, GLP-1 was found to increase cAMP levels in cardiomyocytes, leading to cardioprotective effects [73]. Like GLP-1, GIP also may have vascular protective effects. Recently, elegant work by Nogi et al. shows that GIP inhibits infiltration of macrophages, resulting in decreases in atherosclerotic lesions in both diabetic and nondiabetic mice lacking apolipoprotein E [75]. In Liraglutide Effect and Action in Diabetes: Evaluation of Cardiovascular Outcome Results (LEADER) clinical trial, the effects of GLP-1R agonist liraglutide will be examined for much longer periods [76]. However, even among patients with normal renal function, increases in serum levels of lipase and amylase (reported to be nearly 25% higher than in controls) without symptoms of acute pancreatitis were recognized in this trial [77].

Several studies have shown that DPP-4 inhibitors exhibit cardioprotective effects. Sitagliptin was reported to improve the recovery of left ventricular end-diastolic pressure (LVEDP) in rodents [60, 61]. Furthermore, sitagliptin reduced the size of myocardial infarct area in animal studies [60]. Similar to the effects we reported in the glomerulus [11], the cardioprotective effects are derived from the activation of the GLP-1/cAMP/PKA signaling pathway [60]. Reduced exacerbation of left ventricular function and myocardial dysfunction was reported in DPP-4-null rodents, as compared to wild-type animals, in an induced model of heart failure [58]. Treatment with vildagliptin reduced cardiomyocyte apoptosis and fibrosis in mice, improving the survival rate [62]. In agreement with the outcomes of animal studies, oral administration of DPP-4 inhibitor sitagliptin ameliorated myocardial stunning in response to catecholamine overload in patients with type 2 diabetes and coronary artery disease with normal resting LV function [78]. In contrast to this favorable result, the combination therapy using sitagliptin and granulocyte colony-stimulating factor did not alter the left ventricle ejection fraction (LVEF) at 6-month follow-up (Franz WM et al. presentation abstract; American Heart Association, 2011). Another study using a DPP-4 inhibitor, Saxagliptin Assessment of Vascular Outcomes Recorded in Patients with Diabetes Mellitus-Thrombolysis in Myocardial Infarction 53 (SAVOR-TIMI 53), demonstrated somewhat disappointing results, with more patients hospitalized for heart failure in the saxagliptin group than in the placebo group [79]. The study of the effects of vildagliptin administration demonstrated increased LV end-diastolic and end-systolic volumes (McMurray J. presentation abstract; European Society of Cardiology Annual Meeting, 2013). Similarly, the Examination of Cardiovascular Outcomes with Alogliptin versus Standard of Care (EXAMINE) cardiovascular outcome trial did not observe a reduction in cardiovascular events [80]. In contrast to the relatively short examination period in these studies, Trial Evaluating Cardiovascular Outcomes with Sitagliptin (TECOS) study [59] examined clinical outcomes over much longer periods. Regarding this clinical trial, a recent study reported no increase in admission to hospital for heart failure in the sitagliptin comparing to placebo group [81]. The results were different between SAVOR-TIMI 53 and TECOS; increases in cardiovascular events were recognized in patients with elevated levels of natriuretic peptides, previous heart failure, or chronic kidney disease in SAVOR-TIMI 53, while in TECOS, renal insufficiency patients were excluded. We cannot find any effects of incretin-based therapeutics on macrovascular diseases, but observational period is relatively short in these studies, when compared to UKPDS that showed favorable macrovascular outcomes [1, 2]. Furthermore, the Evaluation of Lixisenatide in Acute coronary syndrome (ELIXA) also showed no increase in admission to hospital for heart failure (Pfeffer MA et al. presentation abstract; American Diabetes Association, 2015). Indeed, it is possible that inhibition of DPP-4 decreases LV function indicated by previous studies, but we have to evaluate these update results described above to clarify the safety of incretin-based therapeutic agents (Table 2).

## 8. Cerebrovascular Effects of Incretin-Based Therapeutic Agents

Previous animal studies showed GLP-1's neuroprotective effects on cerebral ischemia in diabetes. Administration of exendin-4 after cerebral ischemia reduced cerebral infarction area and neurological disorders. As in the case with our results, exendin-4 increased the cAMP/PKA in nerve cells [11], leading to neuroprotective effects. Unlike GLP-1, no study has been carried out about the neuroprotective effects using GIP, though* GIPr* ^−/−^ mice showed impaired learning and neurogenesis [82]. Consistent with GLP-1's neuroprotective effects on cerebral ischemia, the DPP-4 inhibitor, linagliptin, also could reduce ischemic brain damage in diabetic rodents [83]. Further, it seems that the neuroprotective effects are independent from glycemic control and probably mediated by GLP-1, because sulfonylurea glimepiride did not show the same favorable effects though it decreased blood glucose level [83].

## 9. The Effects of Incretin-Based Therapeutic Agents on Diabetic Retinopathy

Diabetic retinopathy (DR) is recognized in a majority of diabetic patients and is one of the major causes of blindness [84, 85]. Chronic hyperglycemic state is a major cause of DR, as supported by the findings of DCCT and UKPDS studies [86, 87]. Also, inflammation and oxidative stress play a significant role in the development of DR [17]. Recent studies suggest that the effects of GLP-1 on the retinal functions could be of significance in the treatment of DR [88]. GLP-1R is expressed in the cells of the retinal ganglion, Müller cells, and pigment epithelial cells [88]. Subcutaneous injection of exendin-4 prevented the decreases in retinal cells and retinal thickness in a rat model of diabetes [88]. Exendin-4 was demonstrated to regulate the Bax/Bcl-2 ratio, leading to neuroprotective effects and prevention of hyperglycemia-induced injury to retinal ganglion cells [89]. Furthermore, intravitreal injection of exendin-4 protected the retina by upregulating the excitatory amino acid transporter expression in the retina [88]. Larger studies, including clinical trials in human patients, are needed to clearly show the potential effectiveness of GLP-1 for DR.

Like incretins, DPP-4 inhibitors also elicit beneficial effects against DR, with oral administration of sitagliptin preventing the pathological changes in the blood-retinal barrier in a rat model of type 2 diabetes [90]. Our recent study showed retinal inflammation to be significantly increased in both diabetes and insulin resistance [17]. Sitagliptin reduced the levels of IL-1*β*, a marker of diabetes-induced retinal inflammation. Furthermore, sitagliptin elicited beneficial effects against the diabetes-induced decrease in circulating CD34^+^ cells, which are enriched in endothelial progenitor cells, compromising the repair process in the damaged vasculature [90]. However, the possibility that the activation of retinal GLP-1R leads to antiapoptotic effects in the retinal endothelial cells could not be excluded, and the actual mechanism and effect remain to be elucidated in further studies in rodents.

## 10. The Effects of Incretin-Based Therapeutic Agents on Diabetic Neuropathy

Chronic hyperglycemic state and impaired insulin signaling induce neurological disorders including peripheral nerve system [91, 92]. It has been reported that the effects of GLP-1 and GIP on the peripheral nervous system could be of significance in diabetic neuropathy. GLP-1 or exendin-4 significantly increased the neurite outgrowth of dorsal root ganglion neurons of rodents [93]. Furthermore, exendin-4 ameliorated the delayed motor and sensory nerve conduction [94]. In diabetic rats, the size of myelinated fibers and the ratio of axon/fiber area were decreased, while administration of exendin-4 significantly ameliorated these disorders [94]. Considering these results, GLP-1 could be a useful treatment for diabetic neuropathy.

## 11. Summary

**Figure 3:**
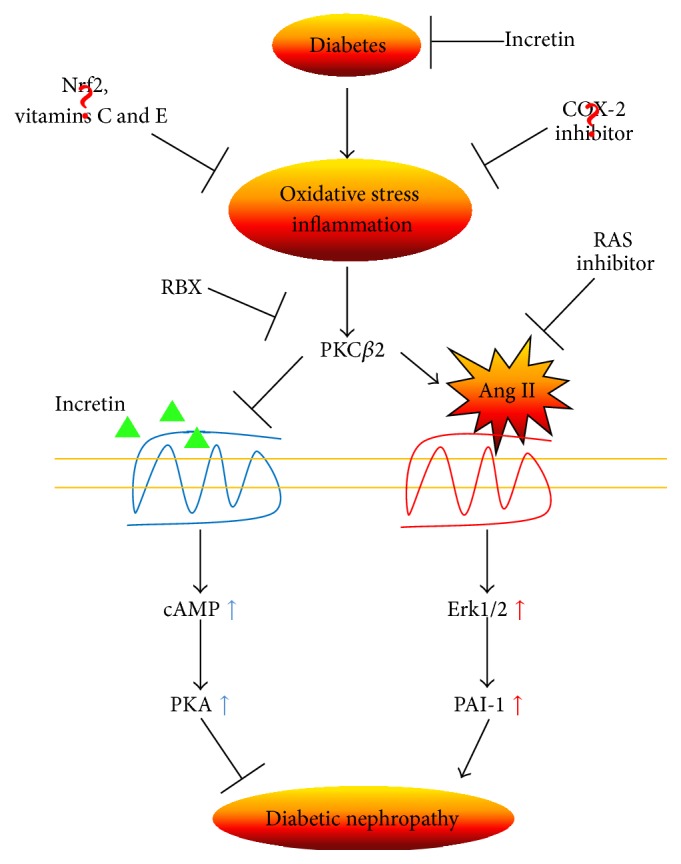
Schematic representation of potential protective factors, including incretin and biological targets of PKC activation that could prevent the progression to diabetic nephropathy. Nrf2, nuclear factor erythroid 2-related factor 2; COX-2, cyclooxygenase-2; RAS, renin angiotensin system; RBX, ruboxistaurin; PKC, protein kinase C; Ang II, angiotensin II; cAMP, cyclic adenosine monophosphate; Erk, extracellular signal-regulated kinase; PKA, protein kinase A; PAI-1, plasminogen activator inhibitor-1.

Both diabetes and insulin resistance induce macro- and microvascular complications. Recent novel therapies for diabetes using incretin or DPP-4 inhibitors elicit biological vasoprotective effects that surpass glycemic control. Incretin-based therapies for diabetic vascular complications show potential as promising for the prevention of diabetic vascular complications, though the activation of PKC*β*2 and angiotensin II could inhibit this therapeutic effect. Different from proposed therapies, such as antioxidant or anti-inflammatory drugs, incretin-based therapies could be beneficial (Figure 3). However, most favorable results appear to be realized in animal disease models. Thus, large-scale clinical trials should be performed to assess the effects of incretin-based treatments on diabetic vascular complications.

## Figures and Tables

**Table 1:** Clinical trials and animal studies of selected incretin-based agents for kidney disease.

Study (drug)	Numbers	Treatment plan	Outcome
Hattori [48] (sitagliptin and alogliptin; 2014)	Sitagliptin and alogliptin; 12	Sitagliptin 50 mg/day for 4 weeks (first period; baseline), alogliptin 25 mg/day for 4 weeks (second period), and sitagliptin 50 mg/day for 4 weeks (third period)	Significant decreases in albuminuria after the change from sitagliptin to alogliptin (81.0 ± 52.4 to 33.9 ± 23.9 mg/g Cr; *P* < 0.05)

Frederich et al. (presentation abstract; American Diabetes Association 74th Scientific Sessions, 2014) Subanalysis study of SAVOR-TIMS53 (saxagliptin; 2013)	Saxagliptin, 2043; placebo, 799	Saxagliptin 2.5, 5, or 10 mg/day (24 weeks)	Significant increases in negative rate of albuminuria (4.6% versus 13.4%)

Groop et al. [53] (linagliptin (MARLINA-T2D); ongoing)	A total of 350 eligible individuals are randomized in a 1 : 1 ratio to receive linagliptin or placebo	Linagliptin 5 mg/day for 24 weeks	

Mima et al. [11] (exendin-4)	STZ-induced diabetic mice + exendin-4; 6 STZ-induced diabetic mice + vehicle; 6	Exendin-4 (1.0 nmol/kg/day) was administrated intraperitoneally for 6 months	Significant decreases in albuminuria (by 27 ± 10%; *P* < 0.05) and mesangial expansion (by 38 ± 10%; *P* < 0.05) compared to DM + vehicle

Park et al. [54] (exendin-4)	*db/db *mice + exendin-4; 8 *db/db *mice + vehicle; 8	Exendin-4 (1.0 nmol/kg/day) was administrated intraperitoneally for 8 weeks	Significant decreases in albuminuria (*P* < 0.01), mesangial matrix fraction (*P* < 0.05), and macrophages infiltration in glomeruli (*P* < 0.01)

Kodera et al. [55] (exendin-4)	STZ-induced diabetic rats + exendin-4; 6 STZ-induced diabetic rats + vehicle; 6	Exendin-4 (10 *μ*g/kg/BW) was administrated intraperitoneally for 8 weeks	Significant decreases in albuminuria (*P* < 0.05), mesangial matrix expansion (*P* < 0.001), and ICAM-1 expression in glomeruli (*P* < 0.01)

Hendarto et al. [56] (liraglutide)	STZ-induced diabetic rats + liraglutide; STZ-induced diabetic rats + vehicle	Liraglutide (0.3 mg/kg/12 h) was administrated with subcutaneous injection for 4 weeks	Significant decreases in albuminuria (*P* < 0.01), NOX4 in glomeruli (*P* < 0.05), and TGF-*β* expression in glomeruli (*P* < 0.01)

Kanasaki et al. [57] (linagliptin)	STZ-induced diabetic mice + linagliptin; 5-6 STZ-induced diabetic mice + vehicle; 7-8	Linagliptin (5 mg/kg BW/day) in drinking water for 4 weeks	Significant decreases in albuminuria (*P* < 0.05) and mesangial matrix expansion (*P* < 0.01)

STZ, streptozotocin; BW, body weight; ICAM-1, intercellular adhesion molecule-1; TGF-*β*, transforming growth factor-*β*.

**Table 2:** Clinical trials and animal studies of selected incretin-based agents for cardiovascular disease.

Study (drug)	Patient numbers	Treatment plan	Outcome
Ku and Su [58] (sitagliptin; 2014)	Sitagliptin, 19; placebo 31	Sitagliptin (100 mg/day) was administrated for 4 weeks	Significant improvement in myocardial function and reduction in postischemic stunning (ejection fraction, 70.5 ± 7.0 versus 65.7 ± 8.0%; *P* < 0.0001; strain rate in ischemic segments, −2.27 ± 0.65 versus −1.988 ± 0.58 s^−1^; *P* = 0.001)

Green et al. [59] (sitagliptin (TECOS); 2015)	Sitagliptin, 839; placebo, 851 (primary outcome)	Sitagliptin 100 mg/day (or 50 mg/day if the baseline GFR was ≥30 and <50 mL per minute per 1.73 m^2^) Median follow-up was 3.0 years	Sitagliptin was noninferior to placebo for the primary compositive cardiovascular outcome (hazard ratio, 0.98; 95% CI, 0.88 to 1.09; *P* < 0.001). Rates of hospitalization for heart failure did not differ between the two groups (hazard ratio, 1.00; 95% CI, 0.83 to 1.20; *P* = 0.98)

Pfeffer et al. (presentation abstract; American Diabetes Association 75th Scientific Sessions, 2015 Lixisenatide (ELIXA); 2015)	Lixisenatide, 3034; placebo, 3034	Lixisenatide 10–20 *μ*g/day Median follow-up was 2.1 years	Lixisenatide was noninferior to placebo for the primary compositive cardiovascular outcome (hazard ratio, 0.97; 95% CI, 0.85 to 1.10). Rates of hospitalization for heart failure did not differ between the two groups (hazard ratio, 0.94; 95% CI, 0.78 to 1.13). Rates of mortality (hazard ratio, 0.94; 95% CI, 0.78 to 1.13)

Ye et al. [60] (Sitagliptin)	Mice underwent coronary ligation + sitagliptin 10; mice underwent coronary ligation + vehicle 10	Sitagliptin (300 mg/kg/day) was administrated by oral gavage for 3 or 14 days	Significant decreases in infarct size (24.3 ± 0.7% in 3 days; *P* < 0.05 and 16.9 ± 0.6%; *P* < 0.001 in 14 days)

Gomez et al. [61]	Hybrid (landrace and large white) pigs with BNP infusion + sitagliptin; 6 Placebo; 6	Sitagliptin (30 mg/kg/BW) was orally administrated for 3 weeks	An increase in stroke volume was observed in the sitagliptin group compared with placebo (+24 + 6% versus −17 + 7%, *P* < 0.01). Glomerular filtration rate declined at week 4 compared with baseline in the placebo group (1.3 + 0.4 versus 2.3 + 0.3 mL/kg/min, *P* < 0.01)

Takahashi et al. [62]	Mice underwent transverse aortic constriction + sitagliptin; 40 Mice underwent transverse aortic constriction + vehicle; 41	Vildagliptin (10 mg/kg/BW day) was administrated by drinking water	Improvement of both LV dilatation and dysfunction in the transverse aortic constriction group ameliorated (*P* < 0.05)

GFR, glomerular filtration rate; BW, body weight; BNP, brain natriuretic peptide.
